# Editorial: Bodies at borders: analyzing the objectification and containment of migrants at border crossing

**DOI:** 10.3389/fsoc.2023.1301780

**Published:** 2023-11-06

**Authors:** Louise Ryan, Izabela Grabowska, Maria Lopez, Vidal Romero

**Affiliations:** ^1^Global Diversities and Inequalities Research Centre, London Metropolitan University, London, United Kingdom; ^2^CRASH, Kozminski University, Warsaw, Masovian, Poland; ^3^Instituto Tecnológico Autónomo de México, Mexico City, Mexico

**Keywords:** global north and south, borders and bordering, asylum seeker, refugees, skilled migrant women, narratives, migration policies

## Introduction

By the end of 2022, the number of forcibly displaced people globally had reached 108.4 million as a result of persecution, conflict, violence, human rights violations, and events seriously disturbing public order (UNHCR, 2023). Efforts to prevent these people from crossing national boundaries have resulted in draconian legislation and the vilification of migrants at various international borders. In the Mediterranean, at the border with “fortress Europe,” there have been thousands of fatalities as migrants risk the treacherous crossing in tiny boats (IMO, 2023). The so-called “weaponization of migration” is apparent in recent events on the Belarussian–Polish border, with hundreds of asylum seekers trapped between rival forces of armed soldiers and subject to “pushbacks” (Guardian Newspaper, 2023). Under the UK government's “hostile environment” policy, many legal immigration routes have been closed, and the rights of asylum seekers have been severely curtailed (Webber, 2019). The so-called “migrant caravan,” which began in Honduras in October 2018, prompted the US and Mexican governments to deploy active-duty military officers to the border, creating more chaos in the area than ever before (Guardian Newspaper, 2022).

Migration, displacement, and border controls are not new, and it can be illuminating to look at previous historical events in order to understand changes and continuities over time. This Research Topic, “*Bodies at the border*,” brings together a range of scholars, including well-established academics and early-career researchers, who present new theoretical approaches, empirical research, and analysis from diverse regions across the world, including the Global South where most migrants and refugees are located.

Using an intersectional lens, our collection of articles explores the complex interplay of diverse aspects of identities including class, age, ethnicity, religion, and gender. In doing so, we seek to advance knowledge on:

- the various policy measures that governments enact to control specific categories of international migration (e.g., Saurombe and Zinatsa; Willers; Sanchez).
- learning lessons from history and previous waves of migration (e.g., Wemyss).
- the use and misuse of a “migrant crisis narrative” (e.g., Grabowska).
- how migrants seek to resist negative representations and discriminatory policies in order to assert their own agency in negotiating national borders (e.g., Ma; Sanchez).
- theoretical and conceptual frameworks that offer new, nuanced understandings of these topics (e.g., López and Ryan).
- the ethical and empirical challenges of researching these topics in contexts that can be risky to both the researchers and participants (e.g., Merlín-Escorza et al.).
- how local populations and organizations react and behave toward “humanitarian tragedies” (e.g., Kyliushyk and Jastrzebowska).

This Research Topic brings together nine articles from around the world in order to examine border crossings in varied contexts and against different policy measures. Moreover, the Research Topic highlights the use of a range of research methods to explore migrants' experiences.

Using critical autoethnography, Ma focuses on her lived experiences as a descendant of forcibly displaced Chinese–Vietnamese people. Her article critically interrogates colonialism and racialization in the Canadian context and how these have produced oppressive policies and practices that have had specific implications for her own family.

Merlín-Escorza et al. use rich ethnographic methods to explore shelter organizations in two countries, the Netherlands and Mexico. While these organizations play a role in protecting the rights of migrants, the article contributes to understanding the fine line between care and control practices in these shelters.

Two articles examine crossings at the Polish border. Kyliushyk and Jastrzebowska use a survey to analyze aid giving and receiving in the context of the mass movement of people from Ukraine to Poland caused by the Russian war on Ukraine, which started in February 2022. The article shows differences between what migrants need and what is offered to them in Poland, addressing both short-term and long-term perspectives. Drawing on a Delphi survey from a large European project, Grabowska focuses in particular on the Belarussian and Ukrainian borders with Poland, with a reference to the so-called “migrant crisis of 2015,” but also explores the positions and dilemmas of stakeholders in treating both refugees in 2015 and Russian war refugees from Ukraine in 2022. She explores the societal dangers of migrant “crises” narratives, including “political functionality” to distract attention away from other kinds of social problems.

Also engaging with narratives, López and Ryan draw on rich qualitative data to analyze the stories of Afghans entering the UK at different periods of time and via varied routes. Building on “journey as a narrative device,” this article uses case studies to explore how migrants tell their stories and present agency, within extremely hazardous situations, to achieve their “imagined futures.”

Also in the UK, Wemyss uses a historically informed lens and notions of “bordering” and intersectionality, as well as archival data, to examine discourses and practices that target seafarers, especially those recruited from the Global South. In doing so, this article analyzes how these seafarers, living and working onboard ships, embody the border in their everyday lives.

Shifting the lens to migratory movements within the Global South, two articles contribute a new understanding to this relatively under-researched field of study. Willers analyzes the inter-connections between anti-smuggling policies and border enforcement through the specific experiences of refugees and migrants, and their intersectional inequalities, in Mexico. Saurombe and Zinatsa examine skilled migration within the continent of Africa and, in doing so, contribute to the literature on labor market integration from the underexplored standpoint of South-to-South mobilities and, thus, advance the understanding of skilled female migrants within the context of family migrations.

Finally, turning to border crossing into the US, Sanchez writes about Mexicans who grew up in the United States without documents. Her article seeks to enhance our understanding of the impacts of changing government policies on vulnerable people, particularly those whose vulnerability is exacerbated by their trust in the government, their fear of the government, or by their exclusion from government programs.

## Conclusion

The articles in this Research Topic highlight the urgency of addressing the following issues and suggest several lines of research for further development.

There is a need for less restrictive and simplified immigration programs in order to ensure the wellbeing and inclusion of migrant communities in several global locations, including the US (see Sanchez) and South Africa (see Saurombe and Zinatsa).

Moreover, there is an urgent need to address the impact of border enforcement and anti-smuggling policies on migrant mobility globally, such as in Mexico (Willers) and the UK. In relation to the UK, our collection of articles highlights the need to re-examine immigration policy from a historical perspective of coloniality, border, and intersectionality (Wemyss) and to reinterpret British migration policy by listening to the migrants' own stories of their migration journeys, including those who have arrived via informal routes (López and Ryan).

Our Research Topic also calls for more attention to be paid to the practices of care and control of migrants in countries of arrival. Kyliushyk and Jastrzebowska highlight the discrepancies between the needs of Ukrainian refugees and the longer-term provision in Poland, while Merlín-Escorza et al. draw attention to shelters in Mexico and the Netherlands as key defined spaces for a growing population in constant mobility.

Finally, we critically interrogate official discourses on migration. Grabowska stresses the need to look closely at the background to the mass arrival of migrants in Poland, in particular the “political functionality” of the migration issue to divert public attention from other problems in the country. Ma calls for a counter-narrative in the context of Canada's asylum policy toward the displaced Chinese–Vietnamese community against the backdrop of the rise of white nationalism, xenophobia, and racism at all levels.

Taken together, these nine articles present new insights into this Research Topic and also suggest important new directions for future research, policy, and practice agendas.

## Author contributions

LR: Writing – original draft. IG: Writing – original draft. ML: Writing – review & editing. VR: Writing – review & editing.

## References

[B1] Guardian Newspaper (2022). Available online at: https://www.theguardian.com/us-news/2022/jun/03/migrant-caravan-tapachula-mexico-biden (accessed October 2, 2023).

[B2] Guardian Newspaper (2023). Available online at: https://www.theguardian.com/world/2023/oct/02/beatings-dog-bites-and-barbed-wire-life-and-death-on-the-poland-belarus-border (accessed October 22, 2023).

[B3] IMO (2023). Available online at: https://missingmigrants.iom.int/region/mediterranean (accessed October 25, 2023).

[B4] UNHCR (2023). Global Trends Forced Displacement in 2023, United Nations High Commissioner for Refugees. Available online at: https://www.unhcr.org/uk/global-trends (accessed October 25, 2023).

[B5] Webber F. (2019). On the creation of the UK's ‘hostile environment'. Race Class 60, 76–87. 10.1177/0306396819825788

